# High fructose corn syrup induces metabolic dysregulation and altered dopamine signaling in the absence of obesity

**DOI:** 10.1371/journal.pone.0190206

**Published:** 2017-12-29

**Authors:** Allison M. Meyers, Devry Mourra, Jeff A. Beeler

**Affiliations:** 1 Department of Psychology, Queens College, City University New York, Flushing, New York, United States of America; 2 Department of Psychology, CUNY Neuroscience Collaborative, City University of New York, New York, New York, United States of America; University of PECS Medical School, HUNGARY

## Abstract

The contribution of high fructose corn syrup (HFCS) to metabolic disorder and obesity, independent of high fat, energy-rich diets, is controversial. While high-fat diets are widely accepted as a rodent model of diet-induced obesity (DIO) and metabolic disorder, the value of HFCS alone as a rodent model of DIO is unclear. Impaired dopamine function is associated with obesity and high fat diet, but the effect of HFCS on the dopamine system has not been investigated. The objective of this study was to test the effect of HFCS on weight gain, glucose regulation, and evoked dopamine release using fast-scan cyclic voltammetry. Mice (C57BL/6) received either water or 10% HFCS solution in combination with ad libitum chow for 15 weeks. HFCS consumption with chow diet did not induce weight gain compared to water, chow-only controls but did induce glucose dysregulation and reduced evoked dopamine release in the dorsolateral striatum. These data show that HFCS can contribute to metabolic disorder and altered dopamine function independent of weight gain and high-fat diets.

## Introduction

Obesity has increased dramatically in recent decades [1], a phenomenon widely associated with the so-called ‘western diet’: energy-dense, highly palatable foods with high fat and sugar content [2]. More recently, there has been an interest in the possible contribution of high fructose corn syrup (HFCS) to the rise in obesity. Used widely in nearly all commercial foods, from bread to beverages [3], HFCS consumption has risen in parallel with increasing body weights and rates of obesity [4]. While evidence suggests links between increased sugar consumption and the rising prevalence of obesity and metabolic disorder [5–7], the contribution of HFCS *per se*, because of its higher fructose content, has been controversial with arguments for [4,8–11] and against [12–15] HFCS constituting a specific liability beyond increased sugar consumption generally.

HFCS-55, containing 55% fructose, 42% glucose, and 3% other saccharides, is primarily used in liquid products [3]. Fructose, including HFCS with its higher fructose content, is more lipogenic compared to other sugars [11,16] and is metabolized differently [17]. Where glucose can enter the cells through GLUT4 (various tissues), GLUT3 (neurons), GLUT2 (homeostasis though uptake in intestine), and GLUT1 (astrocytes and insulin-independent), fructose primarily uses GLUT5, which is not found in pancreatic beta cells, is specific for fructose, and not responsive to insulin [18]. GLUT2 also transports fructose non-selectively, though this low-affinity transporter is involved in transport primarily in the liver, intestine and kidneys [19].

While evidence suggests that fructose [7,11,20,21], and possibly HFCS [22,23] can contribute to the development of metabolic disorder, whether it contributes to weight gain is controversial. Some studies have reported weight gain with fructose or HFCS consumption [23,24] while others have not [21,25,26]. Furthermore, obesity has been associated with altered dopamine (DA) signaling in both human [27–29] and animal studies [30–32]. Reduced dopamine signaling has been suggested to promote compulsive overeating, likening obesity to a dopamine-mediated addiction to food [33,34]. When alterations in dopamine function are observed under high fat diet (HFD), it is difficult to disentangle the potential contribution of increased weight, altered macromolecule composition of the diet *per se*, and effects secondary of metabolic disorder, including altered insulin and leptin signaling and resistance. Here, we examine whether prolonged consumption of HFCS alters dopamine signaling in the dorsal striatum, a region implicated in reinforcement learning, habit and motivated behaviors, including critical for regulating feeding [35–37].

## Materials and methods

### Animals

Male and female C57BL/6 mice (3–4 months at start of feeding protocol) were used for all experiments. Housing for mice was maintained in a 12:12-hour light-dark cycle. All experiments performed during light cycle. All mice were group housed in cages containing 2–4 mice per cage. Mice were not singly housed to avoid inducing stress that could affect both feeding behaviors and dopamine function. All animal experiments were approved by the Queens College Institutional Animal Care and Use Committee in accordance with National Institutes of Health guidelines for the responsible use of animals in research.

### Diet protocol

Mice were placed into either a control group (n = 20) receiving chow diet and water or a high fructose corn syrup (HFCS-chow) group (n = 25) receiving chow diet and a 10% solution of HFCS-55 (55% fructose, 42% glucose, 3% other saccharides, Best Flavors, CA) in their drinking water. Body weight was measured weekly. Water/HFCS was changed and measured twice weekly, total consumption was divided by number of mice in the cage to estimate individual consumption. Sufficient HFCS was available such that it was not a limited, competitive resource. HFCS was kept refrigerated during storage. Food was changed twice weekly, but consumption among group housed mice could not reliably be measured. Though single housing facilitates accurate individual measurement of consumption, it also induces stress, which could alter consumption, metabolism and induce stress-related changes in dopamine and insulin function.

### Glucose challenge

Prior to glucose challenge (at week 15), mice were food and water deprived for 6 hours. Blood glucose was measured with a glucometer (One Touch Ultra 2, Johnson & Johnson) using tail blood. After measuring baseline fasting glucose, dextrose (1 g/kg) was administered via intraperitoneal (i.p.) injection and blood glucose measured at 15, 30, 60, 90, and 120 minutes following injection.

### Fast-scan cyclic voltammetry

Following the glucose challenge at week 15, a subset of HFCS+Chow mice (n = 8) and Chow mice (n = 11) were used for fast-scan cyclic voltammetry (FSCV) and reuptake analysis. Mice were anesthetized with urethane (1.8 g/kg i.p.) and placed into a stereotaxic frame. A carbon-fiber microelectrode (CFMEs, constructed as in [38]) was lowered into the dorsolateral striatum (DLS: AP, +1.1 mm; lat, 2.0 mm; DV, 2.5mm, from the cortical surface) and stimulated dopamine release was measured as described previously [38,39]. Briefly, a bipolar stimulating electrode was lowered into the substantia nigra pars compacta (SNC, AP, -3.16 mm; lat, 0.8 mm; DV, 4.3 mm). A chloride-coated silver wire (Ag/AgCl) reference electrode was implanted contralateral to the CFME and secured with a stainless-steel screw and dental cement. A potential applied to the CFME at -.4 V was ramped up to 1.3 V and back, compared to the reference (Ag/AgCl) electrode, with a scan rate of 400 V/s held at -0.4V between scans. CFMEs were cycled at 60 Hz for 15 minutes and then returned to 10 Hz for ten minutes to stabilize background current. Experimental measurements were made at a scan rate of 10Hz. During stimulation protocols, for each 15s recording, background was digitally subtracted by averaging the background current obtained from ten scans selected from prior to stimulation onset [38]. To optimize dopamine signals, both the CFMEs and stimulating electrodes were systemically lowered in .1 mm increments. At each increment, a train of current (24 pulses, 4 ms per pulse, 60 Hz, 150 μA) were used to evoke a reproducible dopamine release. After optimization, dopamine was evoked using 5 pulses of stimulation administered at 5, 20, and 60 Hz, with 2 minutes between stimulations. A cyclic voltammogram electrochemically identified dopamine with a peak oxidation at .6 V and a reduction peak at .2 V. Following recording, recording site was marked with trypan blue (80 nL) and histology performed to determine placement of working electrodes. The CFMEs were calibrated following the experiment using a micro flow cell and 1 μM dopamine solution.

### Dopamine reuptake

Demon Voltammetry Analysis Software was used to model dopamine reuptake kinetics ([40]; Wake Forest University, Winston-Salem NC). The decay constant tau was used as a measure of dopamine reuptake and area under the curve (AUC) was used as a measure of overall DA signaling. Tau was calculated from an exponential curve fit and is highly correlated with changes in Km (r = .9899), suggesting tau is an accurate measure of DA clearance [40]. The area under the curve was calculated using trapezoid method.

### Statistical analysis

Mixed ANOVAs were used to test for statistical significance for both the glucose challenge (group x sex x timepoint) and FSCV (group x sex x frequency) data. For FSCV data, post-hoc tests were used to determine significant group differences at individual frequencies tested, with Bonferroni correction for multiple comparisons. All statistical analyses were performed using R statistical software (aov, R version 3.2.3 (2015-12-10); The R Foundation for Statistical Computing, http://www.r-project.org).

## Results

### High fructose corn syrup did not increase body weight

**Fig 1:**
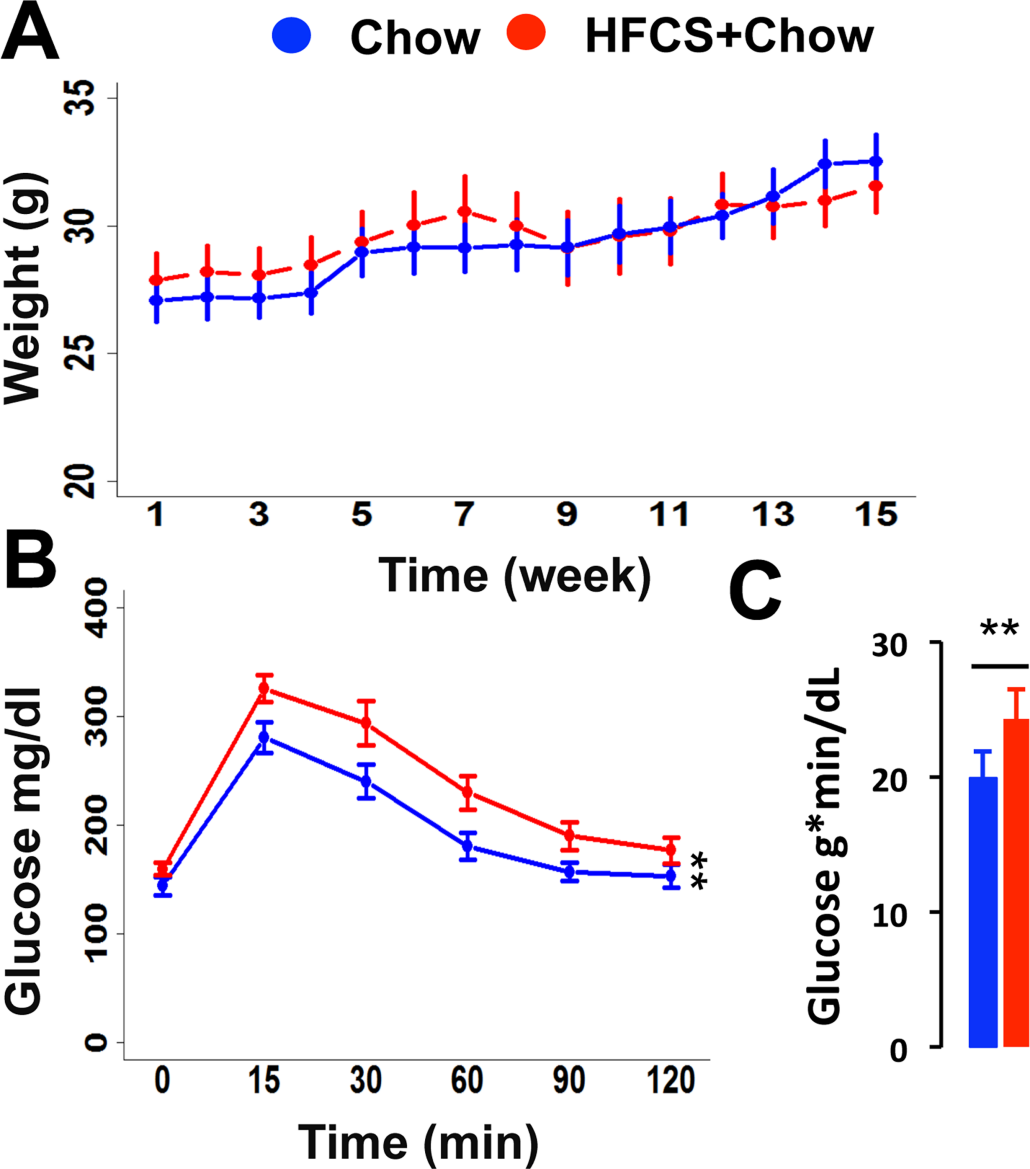
Body weight and glucose challenge. (A) Average weekly body weight across 15 weeks of experiment. (B) Glucose challenge (1 g/kg dextrose) at week 15. (C) Area under the curve for the glucose challenge. N = 20 (chow), 25 (HFCS-chow); ANOVA (panels A/B, repeated measures: group * sex * measurement timepoint; panel C: group * sex), * < .05, ** < .01, *** < .001, error bars S.E.M.

Mice in the HFCS-chow condition consumed significantly more liquid (*F*_(1,11)_ = 82.63, *p* < .001) compared to controls. HFCS-chow mice consumed an average of 13.5 mL/day per mouse of HFCS solution compared to 3.5 mL/day of water consumption in chow mice, contributing approximately 4 kcal to each mouse's daily caloric intake. The HFCS-chow fed mice did not gain more weight compared to chow controls (Fig 1A, *F*_(1,41)_ = .24, *p* = .63). There were no significant sex by group differences (*F*_(1,41)_ = 1.99, *p* = .16) so the male and female data were combined.

### HFCS-chow induced glucose dysregulation

**Fig 2:**
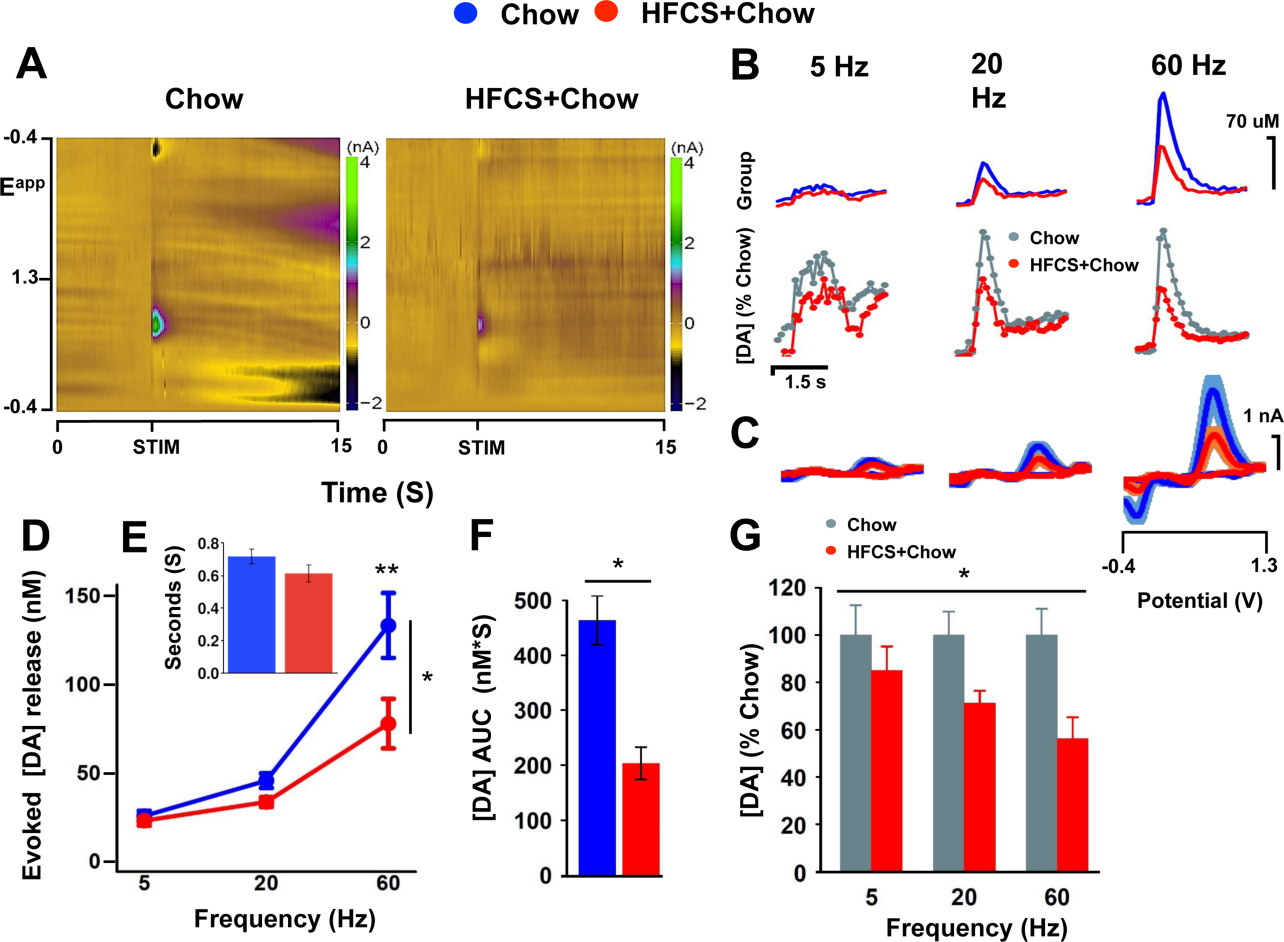
HFCS attenuates evoked dopamine release in the dorsolateral striatum. (A) Average color plots for Chow and HFCS+Chow at 60 Hz, 5 pulses. (B) Average dopamine release by group across frequencies, showing: top, raw data (HFCS, red; Chow, blue); bottom, HFCS group (red) normalized to controls (normalized controls, gray trace). (C) Average voltammograms for Chow (blue) and HFCS+Chow (red) across frequencies. (D) Average peak DA concentration across frequencies. (E) Average tau (decay rate) for Chow (blue) and HFCS-Chow (red) at 60Hz, 24 pulses. (F) Average AUC for Chow (blue) and HFCS-chow (red) at 60Hz, 24 pulses (G) Percent decrease normalized to Chow (gray) across frequencies (red). N = 11/8, Chow, HFCS+Chow, respectively. ANOVA (panels D/G, repeated measures, group * sex * frequency; panels E/F, group * sex), * p < .05, error bars S.E.M.

Glucose levels were tested at week 15. There was no difference in fasting glucose between groups (*F*_(1,41)_ = 2.57, *p* = .12), but the HFCS-chow group exhibited a higher peak glucose and reduced clearance compared to chow controls (Fig 1B, *F*_(1,41)_ = 8.100, *p* < .01), again with no group by sex differences observed (*F*_(1,41)_ = .56, *p* = .46). The mean area under the curve (AUC) for each group is shown in Fig 2C (*F*_(1,41)_ = 8.26, *p* < .01). Though sex differences were not statistically significant, the effects of HFCS on glucose handling appeared more pronounced in males. As our study may be underpowered to reliably detect sex differences, the AUC means for the glucose challenge is shown broken down by diet and sex in Table 1.

**Table 1:** Area under the curve for glucose challenge (at 15 weeks).

Diet	AUC^1^	Sex	AUC^2^
Chow	19.90±2.02	males	24.32±1.60
		females	17.98±0.98
HFCS-Chow	24.30±2.16	males	29.23±2.12
		females	20.43±1.18

^1^ for both sexes combined

^2^ for each sex individually

### HFCS attenuates evoked dopamine release in the dorsolateral striatum

To assess whether HFCS induces impairments in evoked DA release, FSCV was used to measure evoked DA release in the dorsolateral striatum (DLS). A stimulation train of 5 pulses was administered to the substantia nigra pars compacta in descending order at 60, 20, and 5 Hz, with 2 minutes intervals between scans at different frequencies. Evoked peak dopamine release was significantly lower in HFCS-chow mice compared to chow controls (Fig 2A–2D, *F*_(1,15)_ = 8.079, *p* < .05), with reduced responsiveness to increasing frequency compared to controls (group x frequency, *F*_(1,15)_ = 8.167, *p* < .05); that is, the percent decrease in evoked release in HFCS-chow mice relative to controls is greater at higher frequencies (Fig 2G, *F*_(1,15)_ = 5.535, *p* < .05). Post-hoc tests to assess group differences at each frequency tested indicated significant effects of HFCS at 60 and 20 (*p* < .01, .05, respectively), but not at 5 Hz (*p* = .39), suggesting that higher frequency dopamine cell activity, associated with phasic dopamine cell activity, may be more affected than lower frequency, tonic activity.

In the presence of attenuated evoked DA release, prolonged DA clearance may act as a compensatory mechanism to increase DA signaling [41]. Reduced DAT expression and function are often observed in rodent DIO model, both with and without concomitant reductions in released dopamine [42]. We observe no decrease in clearance assessed by tau (time to half peak, Fig 2E, *F*_(1,15)_ = 1.57, *p* = 0.23). To assess total DA signaling we calculated the area under the curve for DA traces. Consistent with reduced peak release, HFCS-Chow show significantly reduced AUC, suggesting a decrease in total dopamine signaling in HFCS-Chow mice relative to chow controls (Fig 2F, *F*_(1,15)_ = 5.96, *p* = .05). Although obesogenic diets have been shown to reduce DAT surface expression/function in the mesolimbic pathway [31,32,43–45], reduced DAT has not been consistently reported in the absence of weight gain [45].

**Fig 3:**
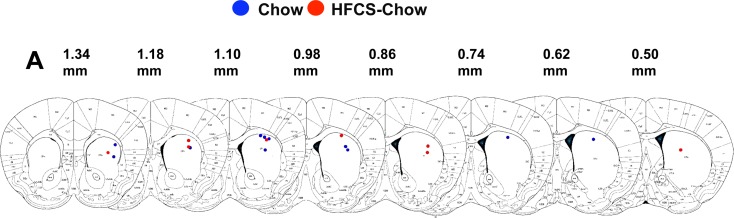
Recording sites for fast scan cyclic voltammetry recording. Blue circles are recording sites for Chow and red circles are recording sites of HFCS+Chow. Chow, n = 11; HFCS-Chow = 8.

Dopamine was electrochemically identified through its voltammogram signature (Fig 2C) and recoding sites were histologically verified (Fig 3). There were no significant sex or group x sex differences in evoked DA release (sex, *F*_(1,15)_ = .513, *p* = .484; interaction, *F*_(1,15)_ = .000, *p* = .993), AUC (Fig 2F, *F*_(1,15)_ = .278, *P* = .606) and uptake kinetics (*F*_(1,15)_ = .459, *p* = .509), thus sexes were combined for analysis and figures.

## Discussion

Evidence suggest that increased consumption of fructose, possibly via HFCS, can induce metabolic dysregulation [7,11,20–23], though whether this is associated with weight gain has been controversial. Here we demonstrate that mice maintained on standard chow with *ad libitum* access to HFCS exhibit altered glucose regulation compared to chow controls in the absence of any significant weight gain. These data indicate that HFCS-induced metabolic dysregulation can arise independently of obesity associated with increased fat consumption. The HFCS induced glucose dysregulation observed here is consistent with previous studies of fructose and HFCS [17,20,21,23]. Several studies have observed metabolic abnormalities with fructose or HFCS in the absence of weight gain. For example, Blakely et al demonstrated that a diet of 15% d-fructose did not significantly alter body weight or food intake, but elevated fasting insulin [46]. Similarly, a study by Huang et al [21] showed that both the HFD and high fructose increased fasting insulin, though only mice on HFD exhibited obesity.

Here we use both males and females, providing evidence in rodents that the effects of HFCS on metabolism and dopamine can be observed in both sexes. Though we observe some indication that the metabolic effects may be more pronounced in males than females (Table 1), these differences were not statistically significant, likely due to a lack of power as our study was not designed specifically to assess sex differences. The human literature on sex differences in fructose-related metabolic effects is complex and varies depending upon the measure (e.g. [47]). With respect to glucose regulation specifically, earlier findings suggested that men were selectively susceptible to fructose-induced glucose dysregulation [48]; however, subsequent studies have found contrary results and suggest that sex differences in response to fructose may be age-dependent [49]. As metabolic studies can be conducted in humans, the primary importance in observing HFCS effects in both sexes here is to lend support to the appropriateness of the rodent model than to make inferences from rodents about sex differences in humans.

The mechanism by which HFCS induces metabolic disorder is likely related to fructose metabolism. Fructose bypasses the phosphofructokinase regulatory step in glycolysis, unlike glucose, and has a rapid uptake into the liver [50]. Fructose also has critical effects on lipid metabolism. The cause for hypertriglyceridemia is proposed to be increased hepatic *de novo* lipogenesis, which leads to hepatic insulin resistance [14,16,51]. This may occur due to increased diacylglycerol, an activator of protein kinase C (PKC) which causes decreased tyrosine phosphorylation of the insulin receptor, increased glucose production, and impaired glucose tolerance [11].

The nigrostriatal and mesolimbic DA pathways are important modulators of feeding behaviors and obesity [36,52–56]. Reduced dopamine function has been consistently associated with obesity, though the exact cause is not clear [57,58]. Three potential causal factors have been identified: diet composition *per se* (increased fat, independent of caloric intake and body weight), insulin resistance associated with obesity and HFD, and increased body weight and adiposity [42,53]. An increased ratio of fat macromolecules in the diet can induce changes in the DA system independent of total caloric intake or body weight [59]; specifically, a downregulation in D2/D3 receptors has been observed, although evoked DA release has not been tested under these conditions. In the present study, DA attenuation was observed with HFCS added to the diet in the absence of increased fat consumption or increased body weight, indicating that neither is necessary for dietary induced changes in DA function.

Our data shows HFCS induces glucose dysregulation in parallel with reduced evoked DA release. Reduced DAT function in response to HFD is one of the most robust findings in the rodent DIO literature. Rodents with access to HFD at 6 weeks or more have slower DA clearance and reduced surface expression of DAT [31,32,43–45]. This altered DA clearance is likely a consequence of insulin resistance [44]. Insulin resistance disrupts PI3K/Akt signaling, known to regulate DAT [31], causing reduced DAT expression on the plasma membrane [60]. Interestingly, despite reduced evoked dopamine release, we did not observe any evidence of altered reuptake kinetics in mice with access to HFCS and observed reduced total dopamine (AUC), suggesting that HFCS is not downregulating DAT, in contrast to high fat diets. This may suggest a different underlying mechanism or reflect an early stage in progressive diminution of dopamine function where increased DAT appears later as a compensatory mechanism.

Finally, we tested HFCS because it is ubiquitous in Western diets [3,6,61]. Moreover, because of differences in metabolic handling of fructose, there have been suggestions that HFCS *per se*, versus other sugars, may particularly contribute to increased obesity [4,8–11,24]. A review of this literature is beyond this scope of this discussion; however, accumulating evidence suggests the metabolic effects of HFCS are likely not substantially different from sucrose, at least acutely [12,13,15,25,62]. This does not diminish the significance of the present data, but does suggest the reduced evoked dopamine observed here might not be unique to HFCS. That said, in comparing access to HFCS vs. sucrose, Levy et al [63] suggest that HFCS has a greater effect in altering reward related gene expression compared to sucrose. While many FSCV studies have examined dopamine activation in response to sugar ingestion (e.g., [56,64,65]), here we assess more global changes in dopamine function after extended access to HFCS, analogous to studies of the effect of high fat diet on dopamine physiology. Future studies might include both HFCS and sucrose to assess whether the increased sugar consumption more broadly, in the absence of weight gain or increased fats, can alter dopamine physiology.

## Conclusion

We confirm prior studies demonstrating that HFCS can induce metabolic dysregulation and add to accumulating data that this can arise in the absence of obesity. Reduced dopamine is associated with obesity (for review, [57]) and may contribute to compulsive eating [33,34,54,66]. We demonstrate that HFCS can impair dopamine function in the absence of weight gain or increased fat consumption. As reduced dopamine function has been implicated in compulsive behaviors [67,68] and reduced energy expenditure [57,69,70] and insulin dysregulation incurs increased obesity risk [71], changes in glucose regulation and dopamine function induced by HFCS may precede and contribute to obesity in the long-run.

Increased consumption of sugar-sweetened soft drinks has been associated with increased rates of obesity and metabolic disorder, especially in developed countries that consume a ‘Western’ diet when compared to those countries with lower access [9]. Although most softdrinks are sweetened with HFCS [3], whether it is HFCS *per se* that contributes to this risk has been controversial [12,13,15]. The addition of HFCS to current rodent DIO models may better recapitulate modern, Western diets associated with increased rates of obesity, allowing for better characterization of both dietary-induced obesity and underlying mechanisms.
